# Accurate Design of Low Backscattering Metasurface Using Iterative Fourier Transform Algorithm

**DOI:** 10.1038/s41598-017-11719-7

**Published:** 2017-09-12

**Authors:** Dan Wang, Zhen Guo Liu, Jie Zhao, Qiang Cheng, Tie Jun Cui

**Affiliations:** State Key Laboratory of Millimeter Waves, Department of Radio Engineering Southeast University, Nanjing, 210096 People’s Republic of China

## Abstract

An accurate method is proposed to design low-backscattering metasurfaces efficiently using an iterative Fourier transform algorithm, which avoids the large amount of time-consuming numerical simulations of complicated electromagnetic problems and provides satisfactory performance to reduce the backward scattering. As an example of the application, a broadband low-backscattering metasurface is designed, fabricated, and characterized. Both full-wave simulation and measured results reveal that the proposed method offers a rapid and efficient tool to manipulate the scattering behaviors of the metasurface, and thus realizes significant scattering reductions.

## Introduction

Low backscattering features from planar or curved apertures, wherein the majority of the backscattering light is dispersed into various directions with a diffusion-like behavior, is greatly beneficial for radar and imaging applications in the microwave, terahertz and optical regime^1-4^. Although significant progress of metamaterial absorbers has been witnessed in the past decade by tailoring the electric and magnetic resonances for complete absorption of incident light in a broad spectrum^5–9^, such functional materials generally lack the ability to manipulate the angular dependent scattering properties as desired, which prompts us to find more efficient methods for controlling the behaviors of light. By combining the concept of optical diffusion with the principle of the generalized “snell’s law”, the low backscattering metasurface is proposed and demonstrated to reduce the backward reflection and engineer the whole scattering properties by use of sub-wavelength resonators of varying dimensions patterned at specific positions of the interface^10–16^. To date, several research works have been reported to design the low backscattering metasurface at microwave or THz frequencies based on the inverse design method, mainly focusing on the bandwidth improvement as well as the efficiency enhancement by taking advantages of various optimization strategies^17–20^.

In this paper, we propose and demonstrate the forward design method of the low backscattering metasurface, which enables fast and precise control of the scattering pattern without the efforts of optimization and the risk of non-convergence, thereby greatly improve the design efficiency and render this method more suitable in engineering than aforementioned ones. From the inverse Discrete Fourier Transform (DFT) relationship between the scattering pattern and the element excitations^16, 21–23^, the Iterative Fourier Transform (IFT) method is introduced for the synthesis of the meta-array patterns by only controlling the reflection phase of each element. To achieve full phase range and good phase linearity, a square ring resonator is employed in our design with almost complete reflectivity around the central frequency. Excellent scattering reduction feature is observed within the predefined frequency bands from the simulation and experimental results. Although the current metasurface is designed to operate at microwave frequencies, the methodology can be readily extended to infrared or optical wavelength range by taking the plasmonic effects of meta-atoms into account.

## Theory and Design

### Design method of low backscattering metasurface

From the antenna theory, the scattering pattern of a planar array made of M × N identical elements can be described as

1 $$F(u,v)=AF(u,v)\,\ast \,E(u,v)$$

in which *AF*(*u*,*v*) is array factor and *E*(*u*,*v*) is the element pattern. The array factor can be easily obtained through the superposition of the contributions of all the elements,

2 $$AF(u,v)=\sum _{m=0}^{M-1}\sum _{n=0}^{N-1}{A}_{mn}\exp [j(mu+nv)]$$

where *u* = *k*_0_ *d*_*x*_sin*θ*cos*φ*, *v* = *k*_0_ *d*_*y*_sin*θ*sin*φ*. *k*_0_ = 2*π*/*λ*_0_ is the wave number in free space, and *A*_*mn*_ is the complex reflection amplitude of the element(m, n). *d*_*x*_ and *d*_*y*_ are periods of the elements in x and y direction respectively. It is clear that Eq. 2 represents a finite Fourier series that relates the reflection coefficients of all the meta-atoms to the array factor of the whole surface, which can be calculated through the IFT method. Let *u* = 2*πp*/*M* and *v* = 2*πq*/*N* in which *p* = 0,1,…*M* − 1 and *q* = 0,1,…*N* − 1, Eq. (2) can be rewritten as

3 $$\begin{array}{c}AF(p,q)=\sum _{m=0}^{M-1}\sum _{n=0}^{N-1}{A}_{mn}\exp (j\frac{2mp\pi }{M})\exp (j\frac{2nq\pi }{N})\\ \,\,\,\,\,\,\,\,\,\,\,\,\,\,\,=\,M\cdot N\cdot {F}^{-1}({A}_{mn}),\end{array}$$

in which *F* ^−1^ denotes the 2-D inverse Fourier transform. This formula is well known and employed in several planar array design and synthesis methods^22, 23^.

During the design of the metasurface, the key essential factor is the suppression ratio in the normal direction, which can be written as

4 $$SR=20{\mathrm{log}}_{10}({F}_{\max }/F)$$

where *F*_max_ is the maximum scattering amplitude of the totally reflective metasurface with uniform phase distributions. *F* is the corresponding scattering amplitude when random reflection phases of the meta-atoms are introduced to reduce the backward reflection. In practical design the suppression of the scattering amplitude around the mainlobe is especially favorable and therefore a threshold *MU* is defined in Eq. (5) to ensure satisfactory low backscattering feature of the metasurface, and the cost function can be expressed as

5 $$SR > MU\quad (u,v)\in {S}_{u,v}$$

in which *S*_*u,v*_ is the angular region of the mainlobe.

**Figure 1:**
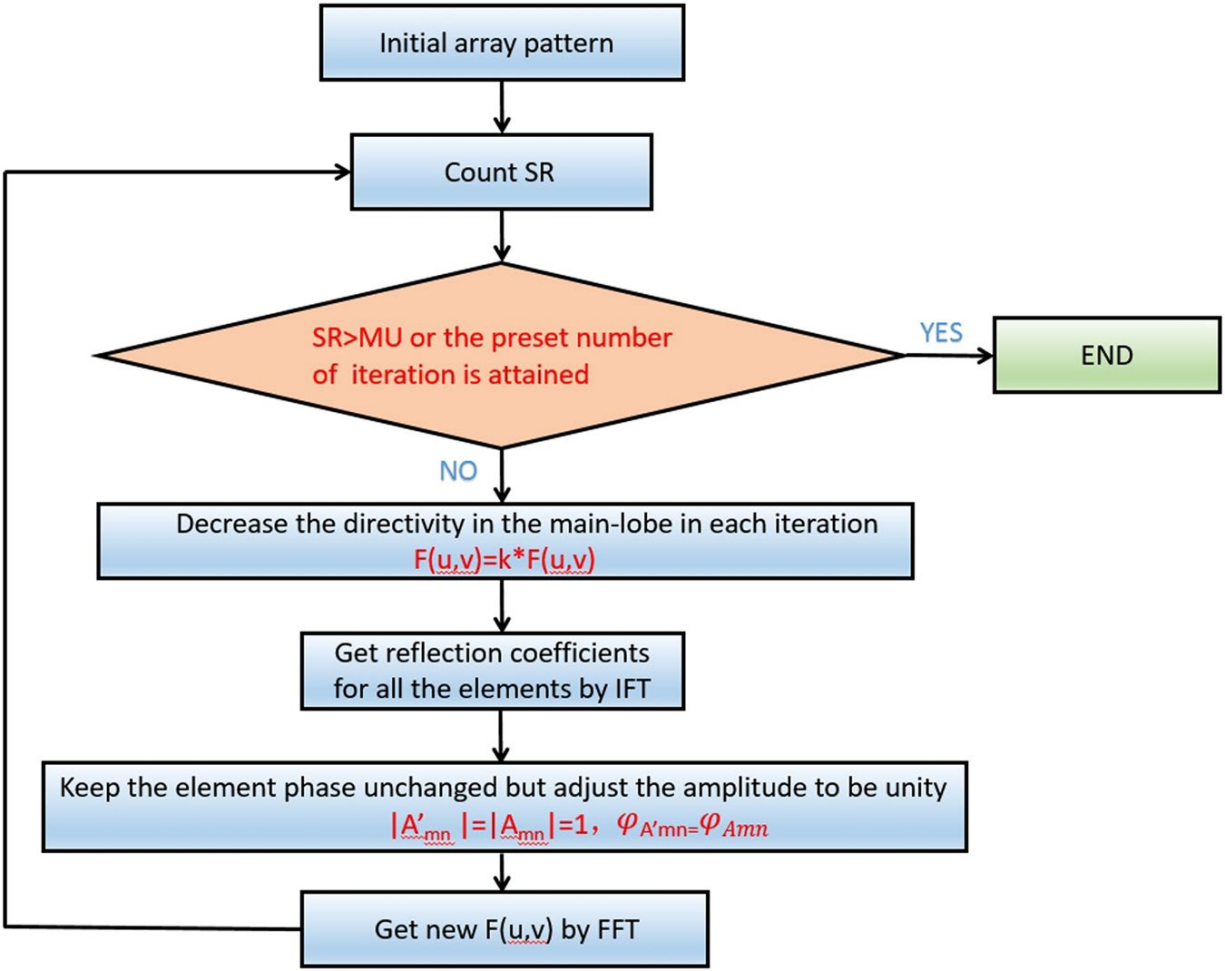
The flowchart for the standard IFT algorithm.

Figure 1 demonstrates the flowchart of the iterative procedure to generate the low backscattering metasurface based on the IFT method. The first step to determine the initial array pattern is to force reflection coefficients for all the elements to be unity, resulting in maximum scattering amplitude $|{F}_{\max }|$ along the normal direction. The calculated directivity will serve as a reference for the following investigations on the reduction of backscattering. Then *SR* is evaluated to verify whether the cost function is satisfied or not. If the suppression ratio in Eq. 4 is less than *MU*, the scattering pattern of the mainlobe should be further revised as

6 $$F(u,v)=kF(u,v)\quad (u,v)\in {S}_{u,v},$$

here *k* is a positive constant smaller than 1 to decrease the amplitude of the scattering pattern around the mainlobe. From Eq. (6) the updated complex reflection coefficients *A*_*mn*_ for all the elements can be obtained from a two dimensional forward Fourier transform (inverse to Eq. 3),

7 $${A}_{mn}=\frac{1}{MN}\sum _{p=0}^{M-1}\sum _{q=0}^{N-1}AF(p,q)\exp (-j\frac{2mp\pi }{M})\exp (-j\frac{2nq\pi }{N}).$$

Since the elements in our design are selected with near complete reflectivity, it offers limited flexibility to tailor the scattering pattern by controlling the phase-only distributions from the meta-atoms. In consequence further modification of the calculated *A*_*mn*_ is particularly required to keep the phase unchanged but adjust the amplitude to be unity,

8 $$\,|{A}_{mn}^{^{\prime} }|=|{A}_{mn}|=1\quad {\phi }_{A{^{\prime} }_{mn}}={\phi }_{{A}_{mn}}.$$

The modified coefficients ${A}_{mn}^{^{\prime} }$ usually result in a new scattering pattern *F*(*u*,*v*) in contrast to the original one, and the *SR* will be recalculated to find out whether the criteria in Eq. (5) has been met.

The iteration in Fig. 1 will be repeated until *SR* is larger than *MU* or the preset number of iteration is attained. It is worth noting that the iteration does not always converge under the given conditions, while the relaxing of constraint (e.g. a smaller *MU*) will be beneficial to improve the stability of the convergence in practice. Although the IFT method provides high accuracy and efficiency in the metasurface design, the whole synthesis is carried out at the central frequency, making it difficult to provide an accurate control of the bandwidth. However, the traditional methods can realize the optimization of the scattering pattern at multiple frequencies for broadband scattering suppression.

### Implementation of Low Backscattering Metasurface

**Figure 2:**
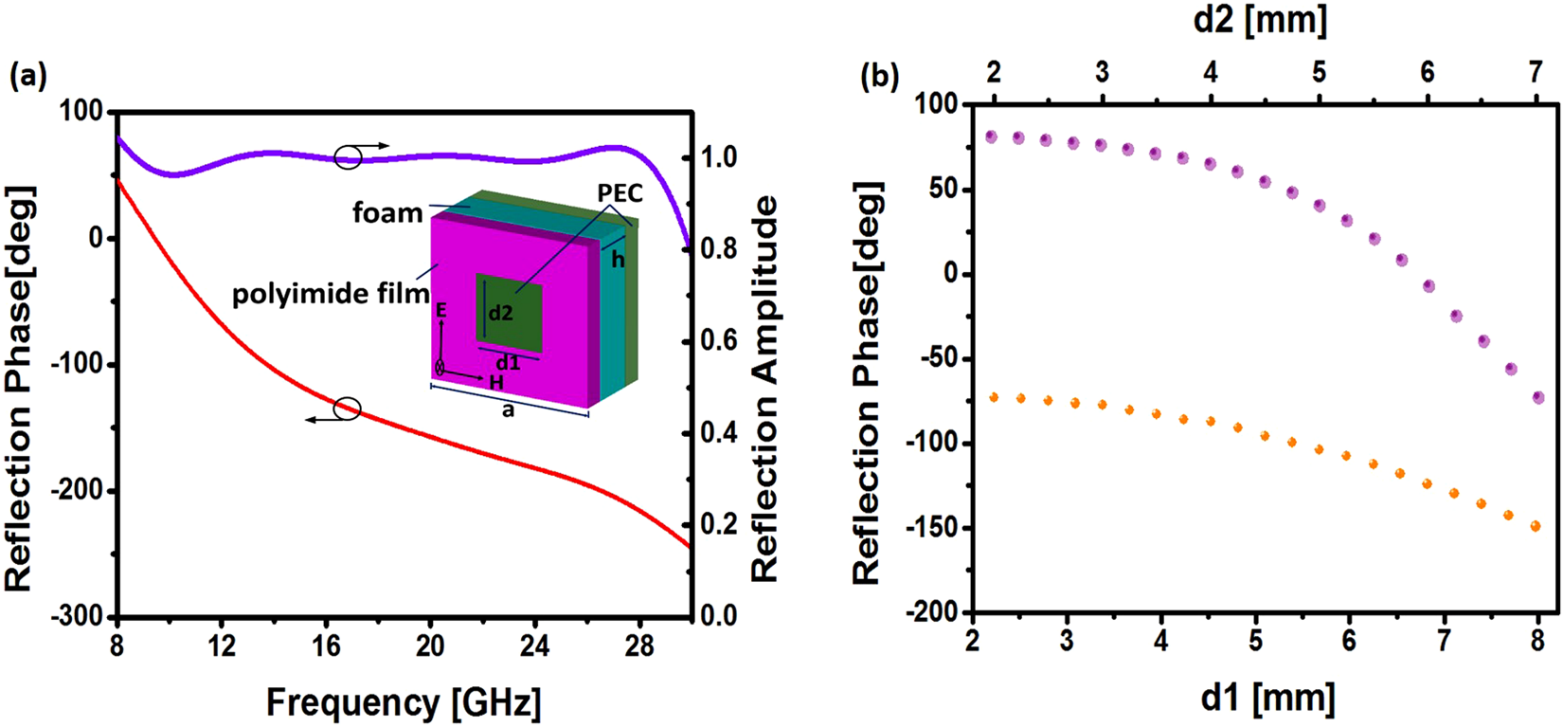
(**a**) Reflection amplitude and phase spectra for the ring resonator. Inset: geometric dimensions of the resonator. (**b**) The reflected phase of the ring resonator as functions of d1 (with a = 14.3 mm and d2 = 1.5 mm) and d2 (with a = 14.3 mm and d1 = 8.5 mm).

So far we have summarized the design procedure based on the IFT algorithm. Next we will focus on the implementation of the metasurface with square ring resonators that are widely used in the metamaterial design. To reduce the weight of the metasurface, the square ring is located at the top of the polyimide film (*ε*_*r*_ = 3.0, tan*δ* = 0.03, h = 0.05 mm) which stands upon a foam substrate (*ε*_*r*_ = 1.1, h = 4.0 mm) with a metallic mirror at the bottom as shown in the inset of Fig. 2(a), leading to entire elimination of transmitted waves at microwave band. We have fabricated the pattern of the meta-atoms on a polyimide film, which is separated by the ground layer with a foam spacer.

Figure 2(a) shows the reflection amplitude and phase for the ring resonator with: a = 14.3 mm, d1 = 6.0 mm, d2 = 2.0 mm, showing good phase linearity, large phase range and high reflectivity as desired. The Floquet’s boundaries are used in the simulation to consider the mutual coupling of adjacent elements, which has been demonstrated to provide enough degree of approximation in the metasurface design^14^. As revealed by previous works, the reflection property of the meta-atom is strongly dependent on the element size and shape. By sweeping the parameters d1 and d2 in the inset of Fig. 2(a), we can establish the relationship between the geometric parameters and the reflection phases for the ring resonator, as illustrated in Fig. 2(b). To guarantee the phase profile responsible for scattering reduction, a number of rings with different sizes should be adapted to accomplish this task. We remark that although the polyimide is a material with relatively large dielectric loss, the reflection amplitude is still close to 1 since most of the energy within the elements is concentrated inside the foam layer and the loss from the polyimide film is very small according to the large contrast of the thickness between the polyimide film and the foam layer.

### Design of metasurface based on IFT

**Figure 3:**
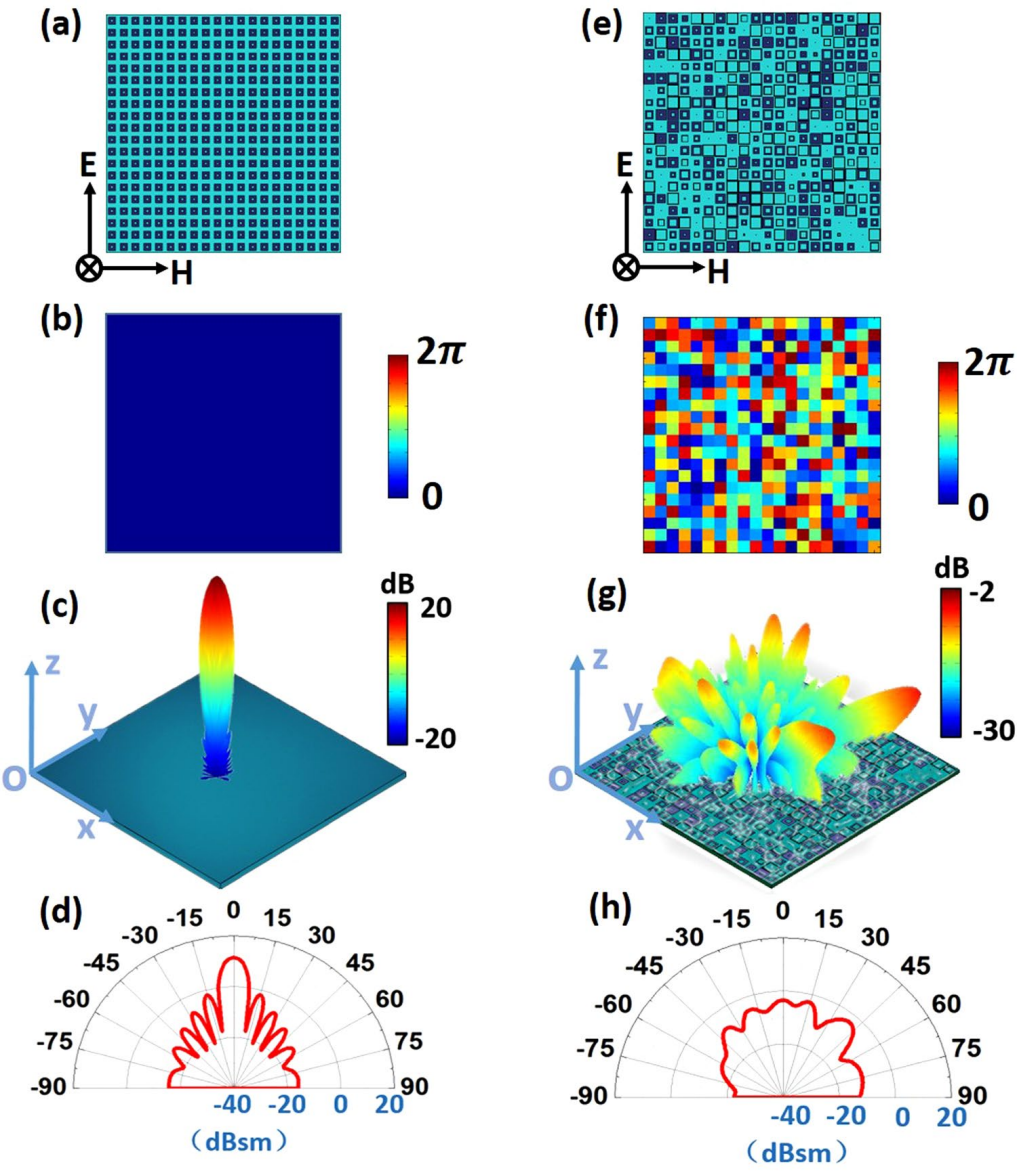
(**a**,**b**) The initial array pattern and the reflection phase distributions of the metasurface composed of square ring resonators. (**c**,**d**) The calculated scattering pattern of the metallic control plate and the corresponding pattern at *yoz* plane. (**e**,**f**) The final array pattern and the reflection phase distributions of the metasurface. (**g**,**h**) The calculated scattering pattern of the synthesized metasurface and the corresponding pattern at *yoz* plane.

As an example, we demonstrate the design process by modeling a feasible implementation of a metasurface constituted by 20 × 20 units. We aim to obtain a metasurface with low backscattering properties at the central frequency 10 GHz. Following the design flow indicating above, we start with an initial pattern by employing the same kind of unit within the surface (Fig. 3(a)) with the phase distributions illustrated in Fig. 3(b), which is characterized by highly directive scattering pattern originating from uniform current distributions excited by the incident waves. During the iteration, *MU* is set to be 10, implying that at least 10 dB suppression of backward scattering will be achieved at normal incidence. After 100 rounds of iterations, we can get the desired phase distributions at the positions (*md*_*x*_,*nd*_*y*_) in Fig. 3(f) from the IFT method, which enables us to determine the element geometries at those locations from the relationship between the geometry parameters and the reflection phase as illustrated in Fig. 2.

As a result, the configuration of the metasurface is eventually achieved by positioning the corresponding elements at the predefined positions (*md*_*x*_,*nd*_*y*_) in the final layout (Fig. 3(e)). The reflected energy is redistributed into various directions within the upper space, yielding a diffusion-like scattering pattern as observed from the numerical simulations. Such phenomenon is closely linked to the destructive interference from waves reflected by the meta-atoms, resulting in dramatic backscattering suppression (more than 10 dB) along the normal of the metasurface by comparing the calculated far field scattering patterns of the metasurface (Fig. 3(g)–(h)) and the metallic control plate of the same size (Fig. 3(c)–(d)) based on Eqs (1–3). The iteration procedure lasts around eight seconds, much shorter than that spent in the reverse design that usually requires complex optimization process^24, 25^, and especially advantageous for the rapid design of low backscattering metasurface in the engineering. Such time cost only refers to the IFT method itself, and the element design and layout generation of the metasurface will additionally cost nearly forty minutes.

### Algorithm Verification

**Figure 4:**
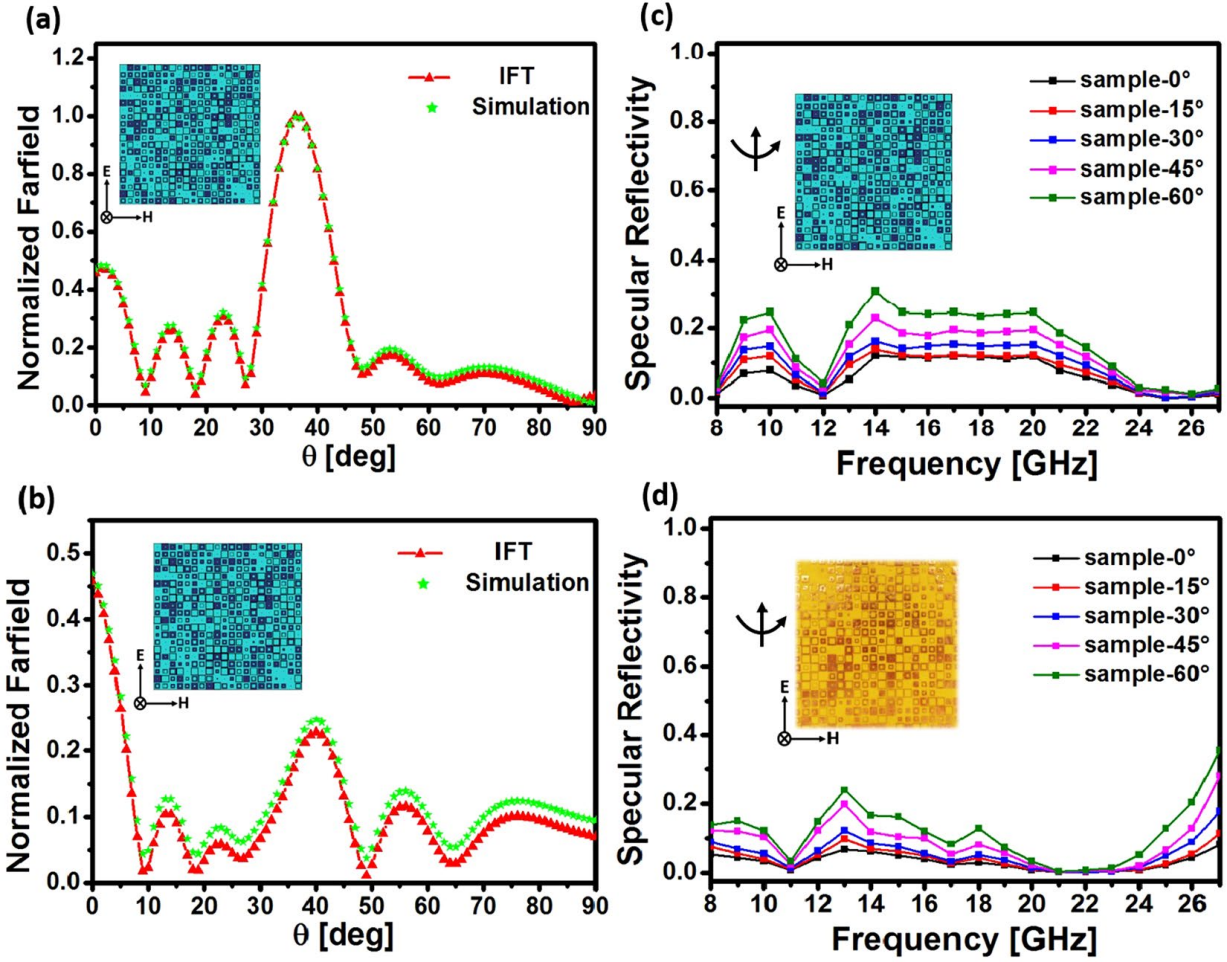
Normalized scattering pattern of the metasurface in Fig. 3(e) at 10 GHz at *xoz* (**a**) and *yoz* (**b**) plane from the IFT method and full-wave simulation. Simulated (**c**) and measured (**d**) specular reflectivity spectra for the designed metasurface with the incidence angle varying from $0^\circ$ to 60°. Inset in (**d**): photograph of the fabricated sample.

The accuracy of the calculation results in Fig. 3 is quantitatively verified through full-wave simulations of the metasurface based on commercial electromagnetic solver (CST Microwave Studio 2014). For simplicity, we only present the simulated and calculated normalized scattering pattern at two mutually orthogonal planes (*xoz* and *yoz* planes) in Fig. 4(a) and (b) for comparison. The excellent agreement between the two results in Fig. 4(a) and (b) demonstrates that the aforementioned fast synthesis model provides a precise and efficient way to manipulate the scattering properties in the desired manner and thus realize a surface characterized by extremely low backscattering features in the microwave and shorter wavelengths.

Figure 4(c) shows the normalized specular reflectivity response of the metasurface plate under the illumination of *y*-polarized plane wave at normal and oblique incidence as a function of frequency. The incident angle is varied from 0° 60° to observe the angular dependence of the suppression feature in Fig. 4(c) and (d), showing that the metasurface suffers from performance degradation for incident angles larger than $45^\circ$. It is clear that the metasurface can operate in a wide spectrum from 9 GHz to 27 GHz with the reflectivity below 0.1 (−10dB), despite the fact that all the elements are designed at the central frequency *f* = 10 GHz. Since the ring resonators exhibits good phase linearity from 8 to 27 GHz in Fig. 2, the relative phase difference between adjacent elements within the metasurface can remain unchanged in a wide spectral range, and thus helps to achieve a wide bandwidth with excellent scattering suppression properties.

## Methods

The reflectivity measurements are performed by the free space method, where a horn antenna is connected with one port of the vector network analyzer (N5230C) to transmit and receive microwave signals. Pyramidal absorbing materials have been placed around the sample to remove the undesired reflections from the surroundings. The sample is placed at a distance of 2.8 m that meets the far-field requirement. In addition, a copper plate of the same size has also been measured for comparison. The transmitting and receiving antennas are placed with various orientations to measure the specular reflectivity with the incidence angle up to 60°.

## Discussion

Using the array pattern described in Fig. 3(e), we experimentally realize the planar metasurface with the standard lithography procedure, as shown in the inset of Fig. 4(d). Figure 4(d) depicts the measured normalized reflectivity for both the metasurface and the control plate from 9 GHz to 27 GHz. As expected from the theoretical predictions, broadband backscattering suppression can be clearly observed at normal incidence, showing excellent agreement with the simulation results in Fig. 4(c) from 9 to 27 GHz. The slight discrepancy between the two results is associated with the fabrication tolerances and experimental errors. However, the overall agreement serves to validate the theoretical calculations and shows the possibility to control the scattering performance of the metasurface to a sufficient accuracy. With the increase of the incident angle, the measured specular reflectivity tends to grow rapidly as demonstrated in Fig. 4(d), consistent with the prediction of simulation results in Fig. 4(c).

## Conlusion

In summary, a method of designing low backscattering metasurface based on the iterative Fourier transform algorithm is proposed and experimental verified in this paper. The method is especially suitable for fast and accurate synthesis of the metasurface with large backscattering suppression. The Iterative Fourier Transform (IFT) method is introduced for the synthesis of the meta-array patterns by only controlling the reflection phase of each element. The time of iteration is much shorter than that spent in the reverse design that usually requires complex optimization process. Good correspondence is demonstrated between theoretically calculated and experimentally measured reflectivity for a metasurface containing 20 × 20 units, showing excellent broadband properties from 9 GHz to 27 GHz at normal incidence and greatly facilitating the related imaging and radar applications in microwave engineering.
